# Impaired neurodevelopment by the low complexity domain of CPEB4 reveals a convergent pathway with neurodegeneration

**DOI:** 10.1038/srep29395

**Published:** 2016-07-06

**Authors:** Jihae Shin, Johnny S. Salameh, Joel D. Richter

**Affiliations:** 1 Program in Molecular Medicine, University of Massachusetts Medical School, Worcester, Massachusetts, USA; 2 Department of Neurology, University of Massachusetts Medical School, Worcester, Massachusetts, USA

## Abstract

CPEB4 is an RNA binding protein expressed in neuronal tissues including brain and spinal cord. CPEB4 has two domains: one that is structured for RNA binding and one that is unstructured and low complexity that has no known function. Unstructured low complexity domains (LCDs) in proteins are often found in RNA-binding proteins and have been implicated in motor neuron degenerative diseases such as amyotrophic lateral sclerosis, indicating that these regions mediate normal RNA processing as well as pathological events. While CPEB4 null knockout mice are normal, animals expressing only the CPEB4 LCD are neonatal lethal with impaired mobility that display defects in neuronal development such as reduced motor axon branching and abnormal neuromuscular junction formation. Although full-length CPEB4 is nearly exclusively cytoplasmic, the CPEB4 LCD forms nucleolar aggregates and CPEB4 LCD-expressing animals have altered ribosomal RNA biogenesis, ribosomal protein gene expression, and elevated levels of stress response genes such as the actin-bundling protein DRR1, which impedes neurite outgrowth. Some of these features share similarities with other LCD-related neurodegenerative disease. Most strikingly, DRR1 appears to be a common focus of several neurodevelopmental and neurodegenerative disorders. Our study reveals a possible molecular convergence between a neurodevelopmental defect and neurodegeneration mediated by LCDs.

Construction of the developing nervous system requires that axons properly synapse on other cells; mostly other neurons but sometimes muscles or gland cells. These tasks rely on actin and microtubule cytoskeleton dynamics in growth cones, the motile structures at the tips of axons and dendrites^1^. Undisrupted temporo-spatial gene regulation in axons and/or growth cones is critical for proper axonal growth, maintenance, and repair during development^2^. Conversely, developmental abnormalities in synaptic compartments can signal onset of neurological disease. For example, spinal muscular atrophy (SMA) is caused by mutation of *SMN1* 3, which is required for survival of motor neurons; reduced SMN1 leads to disturbances in axonal growth and neuromuscular junctions (NMJs) that typically precede motor neuron death^4^,^5^. Schizophrenia (SZ) is another neurodevelopmental disorder associated with axonal abnormalities, which play a role in white matter defects of the disease^6^. Several axon guidance pathway genes such as *ROBO2, SLIT3, PLXNA2* are associated with SZ^7^,^8^ and one SZ candidate gene, *Disrupted-In-Schizophrenia 1*, participates in neurite outgrowth^9^. Improper gene regulation in neurites and/or growth cones also results in neurodegenerative diseases such as amyotrophic lateral sclerosis (ALS). In early stage ALS, nerve terminals and motor neuron junctions are partially degraded while the spinal cord cell bodies are mostly intact^10^. These observations raise the possibility that the disease might begin with distal axonal abnormalities that progress toward the cell body and cause proximal neuronal cell death, described as a “dying-back hypothesis” for neurodegeneration^11^. Thus, understanding molecular mechanism(s) of degeneration in distal motor axons and NMJs might help elucidate late onset motor neuron pathology. Indeed, mutations in the ALS-causing genes *SOD1* and *TDP-43* inhibit neurite outgrowth in cultured neuronal cells^12^,^13^. A newly discovered ALS mutation in *Profillin1* also inhibits axon outgrowth^14^. These observations suggest that neurodevelopmental and neurodegenerative disorders have some features in common, possibly indicating convergent pathogenic pathways.

The CPEB family of RNA-binding proteins regulates mRNA translation through interactions with specific sequences in mRNA 3′ UTRs^15^,^16^. All four CPEB proteins are widely expressed in the brain; two of them, CPEB1 and CPEB3, regulate neuronal synaptic plasticity and learning and memory^17^,^18^,^19^,^20^,^21^. CPEB4 is necessary for hippocampal neuronal cell survival, most likely by regulating mRNA processing required for neuroprotection against cellular stress^22^. The expression of CPEB4 is particularly high in the developing brain and spinal cord across metazoans, indicating an evolutionarily conserved importance of the protein in the developing nervous system. Additionally, CPEB4 is also important in multiple non-neuronal tissues for cancer progression^23^, pathologic angiogenesis^24^ and terminal erythroid differentiation^25^.

The CPEB proteins have carboxy terminal (C-term) RNA binding domains that contain two RNA recognition motifs (RRMs) and two zinc finger (ZF) domains^15^. They also have amino terminal (N-term) regions with little diversity in their amino acid composition, and are hence referred to as low complexity domains (LCDs). In contrast to the RNA binding regions, the LCDs are not well-conserved between CPEB family members, although they do regulate activity of the proteins^26^. For example, the LCD of CPEB1 harbors a critical phosphorylation site that mediates its translational control function by cytoplasmic polyadenylation^27^. The LCD of CPEB3 is particularly glutamine-rich and promotes aggregation of amyloid-like fibers in a similar manner to fungal prions^28^. The role of CPEB4 LCD is not known.

Here we provide a link between the LCD of CPEB4 and neuronal dysfunction during development. Expression of the CPEB4 LCD results in impaired motor axon extension and neurodevelopment, NMJ formation, and mobility defects in embryonic and neonatal mice. LCD-mediated toxicity in spinal motor neurons is associated with an increased stress response, defective ribosomal RNA (rRNA) metabolism, and impaired actin filament organization that perturbs neurite outgrowth. Several of the molecular and physiological readouts caused by CPEB4 LCD mimic those seen in other diseases associated with neurodevelopment as well as neurodegeneration. Our study suggests a possible molecular convergence between impaired neurodevelopment and neurodegeneration that is associated with protein low complexity domains.

## Results

### *Cpeb4* is highly expressed in the nervous system including spinal motor neurons

**Figure 1:**
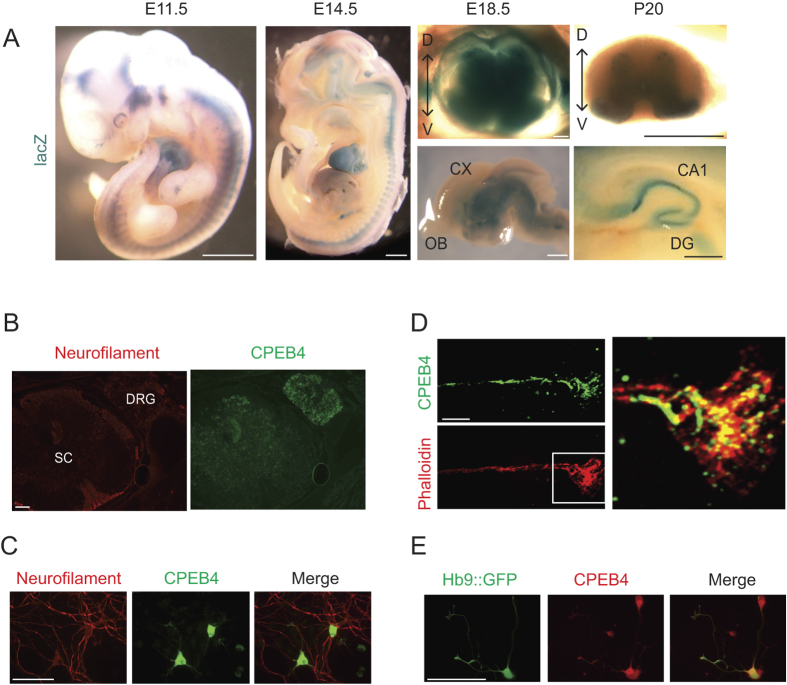
Expression pattern of CPEB4. (**A**) X-gal staining for *Cpeb4-lacZ* expression in *Cpeb4*^GT/+^ embryos or dissected spinal cord (top right) and brain (bottom right) from the indicated developmental stages from embryonic day (e) 11.5 to postnatal day (p) 20. Dorsal (**D**) and ventral (V) portion of the spinal cord is indicated with an arrow. Cortex (CX) and olfactory bulb (OB) are indicated for the sagittal section of the brain (E18.5). CA1 region and dentate gyrus are indicated for the sagittal section of the hippocampus (P20). Scale bars are 1 mm except for E18.5 spinal cord, which is 100 μm. (**B**) Immunohistochemistry of p0 spinal cord (SC) and dorsal root ganglia (DRG) using neurofilament (red) and CPEB4 (green) antibodies. (**C**) Immunocytochemistry of CPEB4 protein (green) in primary neuron cultures co-stained with a neuronal (neurofilament) markers (red). (**D**) Confocal microscopy of an axonal growth cone stained for CPEB4 (green) and phalloidin (red, which denotes filamentous actin). (**E**) CPEB4 (red) immunocytochemistry of primary spinal cord motor neuron cultures from Hb9::GFP (GFP, green) mice. Scale bars are 100μm for (**B**,**C**,**E**), 10 μm for (**D**).

To investigate the functional role of *Cpeb4* in the mammalian central nervous system, we analyzed the expression profile of the *Cpeb4* gene locus during embryonic development by taking advantage of the *lacZ* insertion in the mutant allele of heterozygous gene trap (*Cpeb4*^GT/+^) mice obtained from European Mouse Mutant Archive (EMMA ID:04002). X-gal staining revealed that *Cpeb4* is highly expressed in the nervous system during embryogenesis including brain, spinal cord and the attached dorsal root ganglia (DRG) along with other tissues such as heart (Fig. 1A). At embryonic day (e) 18.5, *Cpeb4* is expressed in the grey matter of spinal cord, which is mainly composed of neurons. The diencephalons as well as the hippocampus, part of the midbrain and the hindbrain also show *Cpeb4* expression. At postnatal day (p) 20, expression in the spinal cord and brain persists including CA1 and dentate gyrus of hippocampus (Fig. 1A). Immunohistochemical staining of frozen sections from p0 spinal cord and DRG with anti-CPEB4 antibody shows the protein to be expressed in these tissues, which are enriched in motor and sensory neurons (Fig. 1B; specificity of antibody demonstrated in^22^,^26^). CPEB4 is expressed in neurofilament (NF)-positive neurons (Fig. 1C) in the cell bodies as well as neurites, suggesting involvement of the protein in neuronal function. Magnified images from immunocytochemical staining of primary spinal cord neuron cultures show that CPEB4 is present in the cell body as well as axons and their growth cones (Fig. 1D). Immunocytochemistry of spinal cord primary neuron cultures from transgenic mice expressing GFP only in motor neurons via the Hb9 promoter^29^ shows that Hb9::GFP-positive spinal motor neurons express CPEB4 (Fig. 1E). Taken together, our data show that CPEB4 is highly expressed in neuronal tissues including brain and spinal cord.

### *Cpeb4* gene trap mice are neonatal lethal with associated motor dysfunction

**Figure 2:**
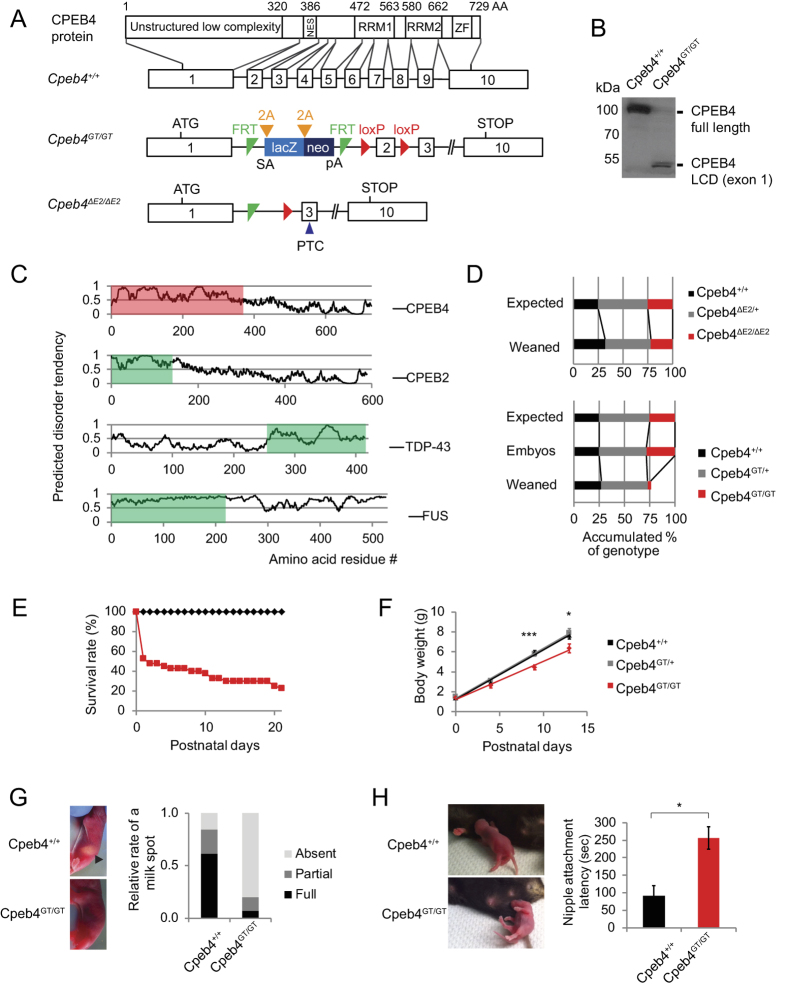
Phenotypes of *Cpeb4*^GT/GT^ mice. (**A**) Schematic diagram of *Cpeb4* WT, *Cpeb4* gene trap and *Cpeb4* exon 2 deletion alleles. White boxes indicate exons of the gene, and the lines indicate introns. For coding regions of the *Cpeb4* WT allele, functional domains of the CPEB4 protein are indicated with amino acid residue numbers on top. NES indicates nuclear export signal; RRM indicates RNA recognition motif; ZF indicates a zinc finger domain. The gene trap cassette contains splice acceptor site (SA) site, *lacZ*, *neo* selection marker gene, and the SV40 polyadenylation sequence (pA). FRT and loxP indicate recombinase sites. 2A indicates a self-cleaving protease sequence. PTC indicates premature termination codon introduced due to the deletion of exon 2. (**B**) Western blot analysis of total brain lysate from *Cpeb4*^+/+^ vs *Cpeb4*^GT/GT^ mice probed with CPEB4 N-term antibody. Full length and the low complexity domain (LCD; 375 residues encoded by exon 1) are indicated. (**C**) Plots for predicted disorder tendencies of CPEB4 (exon 1 is highlighted with red), CPEB2, TDP-43, and FUS; low complexity domains are indicated by green shaded boxes. (**D**) Genotype ratio of the progeny from the *Cpeb4*^ΔE2/+^ mating pairs at weaning age (p21) and the progeny from the *Cpeb4*^GT/+^ mating pairs at e18.5 embryos or weaning age. (**E**) Survival curve of *Cpeb4*^+/+^ (black diamonds) and *Cpeb4*^GT/GT^ (red square) mice after birth (n = 40). (**F**) Body weight of *Cpeb4*^+/+^ (black), *Cpeb4*^GT/+^ (gray), and *Cpeb4*^GT/GT^ (red) animals during postnatal development (shown as mean ± SEM). (**G**) P0 *Cpeb4*^+/+^ and *Cpeb4*^GT/GT^ assayed for milk spot (arrow) incidence. (**H**) Latency of nipple attachment in p0 mice (mean ± SEM). In this and all subsequent figures, one asterisk refers to p < 0.05; two asterisks refer to p < 0.01; three asterisks refer to p < 0.001.

To investigate the functional role of *Cpeb4* in the nervous system, we generated homozygous gene trap (*Cpeb4*^GT/GT^) mice by crossing heterozygous animals (*Cpeb4*^GT/+^). The mutant *Cpeb4* allele contains a gene trap cassette with a strong splice acceptor (SA) sequence followed by *lacZ*, *neo* and a polyadenylation signal (pA)^30^, which generates truncated transcripts that lack the RRMs and ZFs (starting from exon 5) presumably necessary for protein function (Fig. 2A). The mutant transcript is predicted to encode the N-term 375 amino acids of CPEB4 fused to lacZ and neo; however, the self-cleaving 2A peptide between CPEB4 exon 1 and lacZ would co-translationally generate separate protein products. While the robust expression of full-length CPEB4 protein was detected in the wild type spinal cord, exon 1-encoded protein product was readily detected specifically in the *Cpeb4*^GT/GT^ spinal cord with N-term specific antibody for CPEB4 (Fig. 2B) demonstrating that *Cpeb4*^GT/GT^ mice are not only depleted of the full-length but also expressed the mutant form of CPEB4 protein. Little is known about the function of the 375 amino acids encoded by exon 1, but a disorder tendency plot using the IUPred prediction program (http://iupred.enzim.hu) indicates this region (highlighted in red in Fig. 2C), as well as a similar region in CPEB2 (green), to be highly disordered. TDP-43 and FUS, RNA binding proteins linked to the motor neuron disease ALS, also contain disordered domains (green) (Fig. 2C). Disordered regions often serve as hubs for protein-protein interaction networks and are involved in biological processes via modulation of the functions of their binding partners^31^. Disordered regions are often encoded by LCDs, which are comprised of relatively few amino acids. Indeed, 57% of exon 1 of CPEB4 consists of only six amino acids (Supplemental Fig. S1A,B); in comparison, the frequency of these amino acids in the average mouse proteome is ~35%^32^. Moreover, disordered domains have been implicated in a number of human pathologic conditions including Alzheimer’s disease (AD), Parkinson’s disease (PD), and ALS^31^. Therefore, *Cpeb4*^GT/GT^ mice can provide a unique tool to investigate the functional role of CPEB4 LCD *in vivo*.

We also generated a second *Cpeb4* mouse line by deleting exon 2 (Fig. 2A), which would truncate the *Cpeb4* transcript and lead to nonsense-mediated mRNA decay via a premature termination codon (PTC) in exon 3. The exon 2 deletion null mice (*Cpeb4*^ΔE2/ΔE2^) were viable (Fig. 2D, top) and overtly normal. Another group recently described a similar *Cpeb4*^ΔE2/ΔE2^ knockout mouse line that had no discernible phenotype^33^. They performed extensive behavioral tests as well as electrophysiological recordings for synaptic plasticity, which all showed normal responses for *Cpeb4*^ΔE2/ΔE2^ mice^33^. In contrast to the normal phenotype of *Cpeb4*^ΔE2/ΔE2^ mice, we observed substantial neonatal lethality of the *Cpeb4*^GT/GT^ pups. The *Cpeb4*^GT/GT^ mutant line was previously reported to have high mortality within 2 weeks of birth, and the fetal liver had defects in terminal erythroid differentiation^25^; however, the precise cause of the perinatal lethality is unclear and may be attributable to defects in multiple tissues. Genotyping of the pups at weaning (p21) confirmed significant reduction of the *Cpeb4*^GT/GT^ mice at this age (Fig. 2D) in agreement with a previous report^25^. Genotype ratio of the e18.5 embryos is normal, indicating postnatal lethality of the mice. Lethality began soon after birth; 50% died within 24 hours and by 21 days only ~15% survived (Fig. 2E). Strain background of genetically engineered mice is one factor that can influence complicated phenotypes. Even though both *Cpeb4*^GT/GT^ lines used in this study and Hu *et al*.^25^ were initially made in C57BL/6NTac background, they were maintained in C57BLl/6^25^, which was also used for *Cpeb4*^ΔE2/ΔE2^ mice by Tsai *et al*.^33^ (Supplemental Fig. S1C) suggesting genetic background may not be the major reason for the different lethalities of these mice.

We noticed that *Cpeb4*^GT/GT^ animals displayed reduced size and weight compared to wild type littermates (Fig. 2F). In contrast to *Cpeb4*^+/+^ animals, the *Cpeb4*^GT/GT^ mice contained no milk in their stomachs at p0 (Fig. 2G). When quantified as full, partially present, or absent, there was a significant reduction in the milk score in the *Cpeb4*^GT/GT^ pups (Fig. 2G). We also observed that these animals had great difficulty in attaching to the mother’s nipple (Fig. 2H and Supplemental Movie S1). Indeed, a nipple attachment test revealed the *Cpeb4*^GT/GT^ pups took three times longer to successfully perform the task relative to *Cpeb4*^+/+^ pups (Fig. 2H). A more comprehensive analysis of the inability of *Cpeb4*^GT/GT^ in the behavior test revealed profound uncoordinated movements indicative of loss of motor control (Supplemental Movie S1). In addition, we observed occasional gasping of the *Cpeb4*^GT/GT^ pups indicating possible defects in diaphragm function (Supplemental Movie S2). Because *Cpeb4* null mice (*Cpeb4*^ΔE2/ΔE2^) exhibited no overt phenotype, we concluded that the lethality as well as motor neuron defects in *Cpeb4*^GT/GT^ mice resulted from a dominant toxic effect of LCD expression rather than depleting full length CPEB4 proteins. *Cpeb4*^GT/+^ mice were viable and overtly normal (Fig. 2D,F) suggesting mutations from both alleles are required for full penetrance of the phenotype.

### Motor neuron dysfunction in *Cpeb4* ^GT/GT^ mice

**Figure 3:**
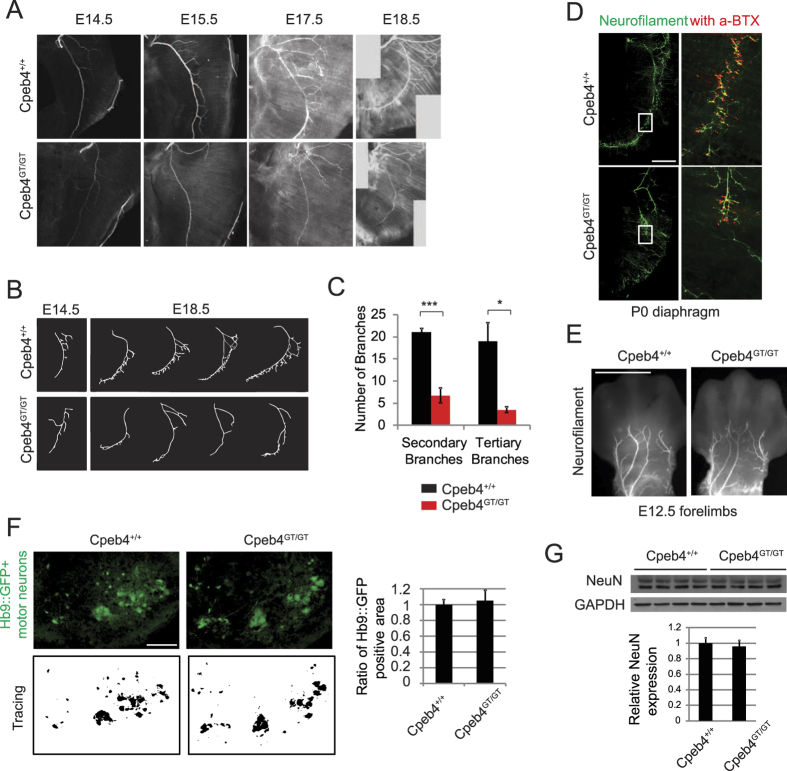
Motor neuron phenotype of *Cpeb4*^GT/GT^ mice. (**A**) Phrenic nerve motor neuron axon tracts in the e14.5 to 18.5 *Cpeb4*^+/+^ and *Cpeb4*^GT/G^ diaphragm stained with neurofilament (NF) antibody. (**B**) Phrenic nerve motor axon tract tracing from E14.5 and E18.5 mice. (**C**) Quantification of the number of branches for e18.5 phrenic nerve from *Cpeb4*^+/+^ (n = 4) and *Cpeb4*^GT/GT^ (n = 4) (mean ± SEM). (**D**) NMJ staining in *Cpeb4*^+/+^ and *Cpeb4*^GT/G^ diaphragm with NF antibody (green) and AF594 conjugated α-bungarotoxin (red). (**E**) *Cpeb4*^+/+^ and *Cpeb4*^GT/G^ E12.5 forelimb sensory neurons stained with NF antibody. (**F**) Immunohistochemistry using GFP antibody for Hb9::GFP positive motor neurons in *Cpeb4*^+/+^ (7 sections from 2 animals) and *Cpeb4*^GT/GT^ (9 sections from 3 animals) spinal cords and the ratio of area for motor neurons (mean ± SEM). (**G**) Western blot analysis of *Cpeb4*^+/+^ and *Cpeb4*^GT/GT^ p0 spinal cords for neuronal marker (NeuN) and GAPDH. Quantification of the relative NeuN level to GAPDH is shown in the histogram (mean ± SEM). Scale bars are 500 μm for (**D**) and 100 μm for (**E**) and (**F**).

Because of the high expression of *Cpeb4* in the spinal cord and the movement deficits in *Cpeb4*^GT/GT^ mice, we suspected that motor neuron development or function might be compromised. To analyze defects in motor neurons, we focused on the phrenic nerve, which originates from the cervical spinal cord and innervates diaphragm muscles. The axon tracts of whole-mount diaphragm were stained with NF antibody, which is previously used to analyze intramuscular motor axon branching in several studies^34^,^35^,^36^. At various stages of development (e14.5 to e18.5), the primary axon branch of the phrenic nerve of *Cpeb4*^+/+^ animals becomes progressively arborized so that by e18.5, numerous secondary and tertiary branches are observed (Fig. 3A, top). In contrast, *Cpeb4*^GT/GT^ axons are thin and display few arbors at e18.5 (Fig. 3A, bottom). Tracing of the axon branches of e18.5 phrenic nerves (Fig. 3B) revealed a significant reduction in the secondary and tertiary axon branching in *Cpeb4*^GT/GT^ mice (Fig. 3C), which may be due to defects in the initiation/growth of the axonal branches or in the management of such structures, or a combination of both. Moreover, co-staining with α-bungarotoxin, a marker for acetylcholine receptors, demonstrated a reduction of NMJ formation in these animals (Fig. 3D), which likely explains their movement defects and failure in nipple attachment. Quantification of α-bungarotoxin intensity over the diaphragm muscle area showed more than ~50% reduction in *Cpeb4*^GT/GT^ mice (Supplemental Fig. S2A) similar to the loss of axonal terminals, indicating reduced innervation and subsequent loss of acetylcholine receptor cluster formation. Neither forelimb nor ophthalmic sensory neurons presented axon branching defects (Fig. 3E and Supplemental Fig. S2B), suggesting that motor neurons are primarily affected by *CPEB4* LCD expression. We expect that the branching defects of the *Cpeb4*^GT/GT^ mice are likely to be attributable to neuronal defects because skeletal muscle tissues did not express CPEB4 protein shown by western blot analysis (Supplemental Fig. S2C).

To assess whether motor neurons in the spinal cord undergo degeneration, we crossed *Cpeb4*^GT/GT^ mice with Hb9::GFP transgenic mice^29^. Immunohistochemistry of cervical spinal cords from Hb9::GFP transgenic mice showed no discernible difference in the area for GFP-positive motor neurons of the p0 *Cpeb4*^+/+^ and *Cpeb4*^GT/GT^ mice, suggesting the defects are specific for axon branching rather than overall cell death (Fig. 3F). In addition, expression level of a neuronal protein, NeuN, measured by western blot analysis was comparable in the p0 *Cpeb4*^+/+^ and *Cpeb4*^GT/GT^ spinal cords, which also suggests lack of cell death (Fig. 3G). However, it is possible that distal motor axon abnormalities precede the ultimate cell death, which was not captured in the time frame of our assay due to the lethality of *Cpeb4*^GT/GT^ mice at p0.

We also tested the possibility that aged *Cpeb4*^GT/GT^ mice may exhibit motor neuron degeneration. About 15% *Cpeb4*^GT/GT^ animals survive to adulthood (Fig. 2E) and become indistinguishable from their *Cpeb4*^+/+^ littermates. Measurement of compound muscle action potential (CMAP), motor unit number estimation (MUNE), motor unit (MU) size and electromyography (EMG) score in sciatic nerve of hind limb of 2 year old *Cpeb4*^GT/GT^ mice did not show any statistical difference, indicating absence of detectable motor neuron degeneration (Supplemental Fig. S2D). Histological analysis of the skeletal muscles showed no visible pathological changes in the *Cpeb4*^GT/GT^ mice (Supplemental Fig. S2E), in agreement with our electrophysiological analysis, and indicates a lack of motor neuron degeneration in adult *Cpeb4*^GT/GT^ mice.

### Low complexity domain-mediated toxicity is associated with nucleolar stress

**Figure 4:**
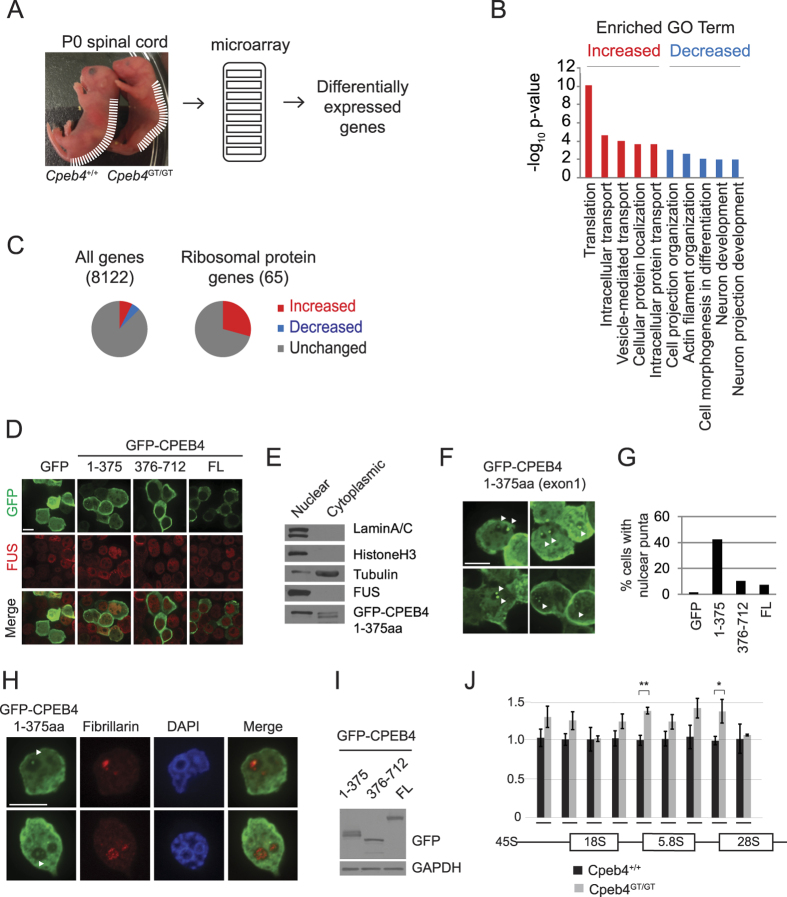
Disordered *Cpeb4* exon 1 LCD induces nucleolar stress. (**A**) Schematic diagram of the analysis to identify differentially expressed genes in *Cpeb4*^+/+^ and *Cpeb4*^GT/GT^ p0 spinal cords. Total spinal cord RNAs were used for genome-wide microarray analysis. (**B**) Gene ontology analysis of differentially expressed genes shown with −log_10_ p-value. The transcripts denoted in red are those that increased the most and those in blue decreased the most in *Cpeb4*^GT/GT^ spinal cord. (**C**) Microarray analysis of differentially expressed genes in p0 *Cpeb4*^GT/GT^ versus *Cpeb4*^+/+^ spinal cords for all or ribosomal protein mRNAs. (**D**) N2a cells transfected with GFP, GFP-CPEB4 residue 1–375, GFP-CPEB4 376–712, or GFP-CPEB4 full length (FL) were stained with GFP and FUS (nuclear protein) antibodies. (**E**) GFP-CPEB4 1–375 expressing N2a cells were fractionated into nuclear and cytoplasmic compartments subjected to western blot analysis probed with the indicated antibodies. (**F**) Magnified images of GFP-CPEB4 1–375 transfected N2a cells stained with GFP antibody. The arrowheads denote nuclear/nucleolar puncta. (**G**) The histogram for percentage of transfected cells with nuclear puncta expressing GFP only (n = 68), GFP-CPEB4 1–375 (n = 104), GFP-CPEB4 376–712 (n = 114), and GFP-CPEB4 FL (n = 96). (**H**) 293T cells transfected with GFP, GFP-CPEB4 residue 1–375, GFP-CPEB4 376–712, or GFP-CPEB4 full length (FL) were stained with GFP and fibrillarin (nucleolar marker protein) antibodies. (**I**) Western blot analysis of the lysates from 293T cells transfected with GFP-CPEB4 residue 1–375, GFP-CPEB4 376–712, or GFP-CPEB4 full length (FL) with GFP or GAPDH antibodies. (**J**) rRNA processing in p0 *Cpeb4*^GT/GT^ (gray bar) *Cpeb4*^+/+^ (black bar) spinal cord from 3–9 animals analyzed by quantitative RT-PCR (Mean ± SEM). Beneath the histogram are the approximate locations of primers for detecting 45S, 18S 5′ junction, 18S, 18S 3′ junction, 5.8S 5′ junction, 5.8S, 5.8S 3′ junction, 28S 5′ junction, and 28S rRNA. Scale bars are 10 μm.

To investigate the molecular lesion(s) caused by CPEB4 LCD, we performed microarray analysis with p0 spinal cord from *Cpeb4*^+/+^ or *Cpeb4*^GT/GT^ mice to identify differentially expressed genes (Fig. 4A). Gene ontology (GO) analysis of the microarray data revealed that transcripts involved in translation, transport, and protein localization were increased whereas mRNAs implicated in cell projection organization and neuron development were decreased (Fig. 4B) in the spinal cord of *Cpeb4*^GT/GT^ mice, which is consistent with the axon branching defects in the *Cpeb4*^GT/GT^ mice (Fig. 3A–D). Of the 8122 RefSeq genes detected above threshold, 1022 (12.58%) were differentially expressed in *Cpeb4*^GT/GT^ tissue. Among these, 613 (7.55%) were increased and 405 (5.04%) were decreased (Fig. 4C). Interestingly, for 65 mRNAs encoding ribosomal proteins, 29% were increased but none were decreased in *Cpeb4*^GT/GT^ spinal cord (Fig. 4C), suggesting CPEB4 LCD may disrupt cellular metabolism related to ribosomal function.

To investigate the cellular function of CPEB4 exon 1 LCD, we expressed a GFP tagged version of this 375 amino acid polypeptide as well as the C-term region (residues 376-712) and the full-length (FL) CPEB4 protein in N2a mouse neuroblastoma cells (Fig. 4D–G) or 293T cells (Fig. 4H–I). At steady state, full-length CPEB4 is predominantly cytoplasmic, even though it shuttles between nucleus and cytoplasm and congregates in the nucleus upon cellular stress^22^. Both GFP-CPEB4 376-712 and GFP-CPEB4 FL were found exclusively in the cytoplasm whereas GFP-CPEB4 1-375 resided in nuclei as well as the cytoplasm partially co-localizing with nuclear protein FUS (Fig. 4D, see Supplemental Fig. S3 for magnified images). The CPEB4 exon 1 LCD is truncated upstream of the nuclear export signal (NES)^22^ (also see Fig. 2A for location of the NES), which likely accounts for its nuclear localization. Subcellular fractionation of the transfected cells showed that GFP-CPEB4 LCD was predominantly nuclear (Fig. 4E) agreeing with immunocytochemistry data. Interestingly, nuclear GFP-CPEB4 1-375 migrates slower than the cytoplasmic proteins (a doublet band in Fig. 4E), which may result from a different post-translational modification such as phosphorylation. We also noticed occasional nuclear puncta in the CPEB4 LCD-expressing cells (Fig. 4F, white arrowheads) with higher frequency compared to GFP-CPEB4 C-term region or GFP-CPEB4 FL (Fig. 4G). To investigate subcellular localization of the GFP-positive puncta in GFP-CPEB4 1-375 expressing cells, we co-stained the cells with a nucleolar marker, fibrillarin (Fig. 4H). Some of the CPEB4-LCD puncta were DAPI-negative and fibrillarin-positive indicating nucleolar localization (Fig. 4H, arrowheads). The expression levels of the GFP-tagged CPEB4 proteins (1–375, 376–712 or FL) were comparable when microscopically examined (Fig. 4D,F,H) and when they were analyzed using GFP antibody with western blot (Fig. 4I). Because nucleoli are sites for ribosome synthesis and assembly, we suspected *Cpeb4*^GT/GT^ mice may have impaired nucleolar function caused by CPEB4 LCD. We analyzed rRNA processing in p0 spinal cord from *Cpeb4*^GT/GT^ mice as readout for nucleolar dysfunction. Quantitative RT-PCR with primers specific for either the junctions or mature forms of rRNA revealed that *Cpeb4*^GT/GT^ had an ~40% increase in the products for 5′ junctions of 5.8S and 28S rRNAs, confirming that rRNA processing was reduced in these mice although mature levels of 18S, 5.8S, and 28S were unaffected (Fig. 4J) suggesting a mild impairment of nucleolar function.

Many RNA-binding proteins localize to various nuclear puncta in eukaryotic cells^37^. Interestingly, the repeat-associated non-AUG (RAN) translation products glycine:arginine (GR_n_) and proline:arginine (PR_n_) dipeptide repeat polymers from the *C9orf72* hexanucleotide repeat expansion, which are low complexity by nature and a major known cause of ALS, are also found in nucleoli where they impede RNA biogenesis and increase ribosomal protein mRNA levels, which may be one cause of cell death^38^. Therefore CPEB4 LCD seems to share a pathogenic mechanism with other LC peptide-related diseases. We speculate that RAN polymer induced toxicity is not only restricted to neurodegenerative diseases, but may also represent a more general phenomenon of low complexity peptide-mediated pathogenesis during neurodevelopment including CPEB4 LCD toxicity in the *Cpeb4*^GT/GT^ mice. Furthermore, nucleolar stress presented by nucleolar aggregates and rRNA processing defects may be specific examples of such a convergent pathogenic pathway caused by low complexity peptide/proteins.

### An actin binding protein DRR1 is commonly upregulated in neurological disorders

**Figure 5:**
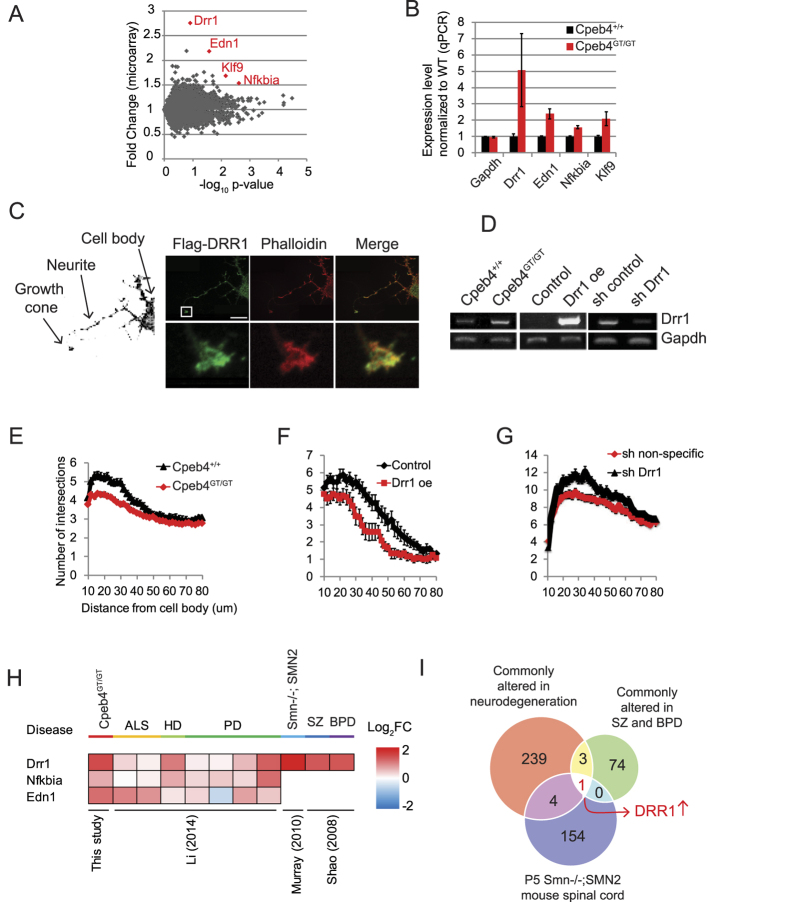
Actin binding protein DRR1 level is elevated in *Cpeb4*^GT/GT^ (**A**) Microarray analysis of differentially expressed genes in p0 *Cpeb4*^GT/GT^ versus *Cpeb4*^+/+^ spinal cords for all detected mRNAs. All 8122 detected RNAs were plotted as fold change (ordinate) in *Cpeb4*^GT/GT^ versus *Cpeb4*^+/+^ compared to −log_10_ p-value (abscissa). Transcripts for *Drr1*, *Edn1, Klf9*, and *Nfkbia* are highlighted in red. (**B**) Quantitative RT-PCR of *Drr1*, Edn1, *Klf9*, and *Nfkbia* RNAs normalized to *Ppia* mRNA from *Cpeb4*^+/+^ and *Cpeb4*^GT/GT^ spinal cord (mean ± SEM). (**C**) Mouse hippocampal neurons expressing ectopic FLAG tagged DRR1 are stained with FLAG antibody (green) or phalloidin (red, which denotes filamentous actin). Insets (bottom) are magnified images of a growth cone. Image on the left side is a black/white image of the same neuron to indicate cell body, neurite and growth cone of the neuron. Scale bar is 20 μm. (**D**) RT-PCR for *Drr1* expression in DIV 4-7 hippocampal neurons from *Cpeb4*^+/+^ and *Cpeb4*^GT/GT^ mice, or neurons expressing ectopic *Drr1*, a scrambled shRNA, or an shRNA directed against *Drr1*. (**E**) Sholl analysis to determine neurite branching in hippocampal neurons from *Cpeb4*^+/+^ (n = 76) vs *Cpeb4*^GT/GT^ mice (n = 111) (mean ± SEM). (**F**) Sholl analysis of WT (n = 15) or neurons expressing ectopic *Drr1* (n = 12). (**G**) Sholl analysis of neurons expressing scrambled (n = 43) or *Drr1* shRNAs (n = 36). (**H**) Heat map for expression level of *Drr1, Nfkbia* and *Edn* mRNAs in *Cpeb4*^GT/GT^ spinal cord and various neurological disorders. Heat map colors correspond to log_2_ fold changes compared to WT animals or control groups and original research article is indicated for each analysis. (**I**) Venn diagram to indicate commonality of regulated transcripts among three studies. 243 commonly altered genes in neurodegeneration by Li (2014) (ALS, HD, PD), 78 commonly altered genes in SZ and BPD by Shao^47^, and 159 differentially expressed genes in p5 spinal cord of Smn−/−; SMN2 mouse (fold change > 1.5) by Murray^48^ were compared for overlapping genes.

To elucidate the molecular mechanism of CPEB4 LCD-mediated toxicity, we further analyzed the differentially expressed genes in p0 *Cpeb4*^GT/GT^ spinal cord. We noticed that several stress response genes such as *Drr1* (down-regulated in renal cell carcinoma 1, also called family with sequence similarity 107 (*Fam107A*)), *Edn1*, *Klf9*, and *Nfkbia*^39^,^40^,^41^,^42^ were significantly increased in *Cpeb4*^GT/GT^ spinal cord (Fig. 5A), which was independently validated by RT-qPCR (Fig. 5B). In contrast, the expression levels of these transcripts were unchanged in the p0 *Cpeb4*^ΔE2/ΔE2^ spinal cord (Supplemental Fig. S4) suggesting these molecular readouts resulted from a LCD expression rather than depleting full length CPEB4 proteins. Among these transcripts, *Drr1* was of special interest because it is an actin binding protein that modulates actin-dependent neurite outgrowth^42^, and thus could be directly relevant to the *Cpeb4*^GT/GT^ mouse phenotype. *Drr1* mRNA is highly and specifically expressed in various human neuronal tissues (Supplemental Fig. S5A). To investigate neuronal expression and subcellular localization of DRR1, we expressed FLAG-tagged DRR1 in hippocampal neurons via lentivirus. Ectopically expressed DRR1 is localized not only in the cell body but also in neurites and growth cones, co-localizing with filamentous actin denoted by phalloidin (Fig. 5C). *Drr1* mRNA is regulated by various stresses including treatment with a glucocorticoid agonist, food deprivation, and social stress^43^. Overexpression of *Drr1* not only inhibits neurite outgrowth *in vitro* but also alters related social behavior *in vivo*^44^.

To test the link between CPEB4 LCD-mediated toxicity and *Drr1* regulation, we turned to cultured mouse hippocampal neurons for ease of use, which like p0 spinal cord have elevated *Drr1* mRNA levels in *Cpeb4*^GT/GT^ compared to *Cpeb4*^+/+^ neurons (Fig. 5D). Sholl analysis of neurite branching shows that *Cpeb4*^GT/GT^ neurons have reduced outgrowth compared to *Cpeb4*^+/+^ neurons (Fig. 5E). Ectopic expression of *Drr1* in *Cpeb4*^+/+^ neurons inhibited neurite outgrowth (Fig. 5F)^42^, while depletion of *Drr1* in *Cpeb4*^GT/GT^ neurons partially rescued the reduction in neurite outgrowth (Fig. 5G). These data suggest that the actin binding protein DRR1 is a downstream factor in CPEB4 LCD-mediated toxicity of neurons, and may contribute to the neuronal dysfunction of the *Cpeb4*^GT/GT^ mice.

*Drr1*, together with *Nfkbia*, is one of 243 commonly altered genes identified using 1,270 post-mortem tissues from 13 patient cohorts for four neurodegenerative diseases including ALS, PD, and Huntington’s disease (HD) (Fig. 5H and Supplemental Fig. S5B)^45^. Another gene whose expression is altered in *Cpeb4*^GT/GT^ spinal cord, *Edn1* is also increased in ALS patients (Fig. 5H)^46^. Moreover, *Drr1* is one of 78 significantly altered genes in cortical tissues of 29 bipolar disorder (BPD) and 32 schizophrenia (SZ) patients^47^. It is also one of 159 altered genes in p5 spinal cord of a mouse model (*Smn*−/−; SMN2) for SMA^48^ (Fig. 5H). We compared the 243 commonly altered genes in neurodegeneration identified by Li, and the 78 commonly altered genes in BPD and SZ identified by Shao together with the 159 altered genes in p5 spinal cord of the mouse model for SMA^48^. *Drr1* was the only gene alteration shared by the three different studies mentioned above (Fig. 5I) suggesting its importance for neurological disease.

**Figure 6:**
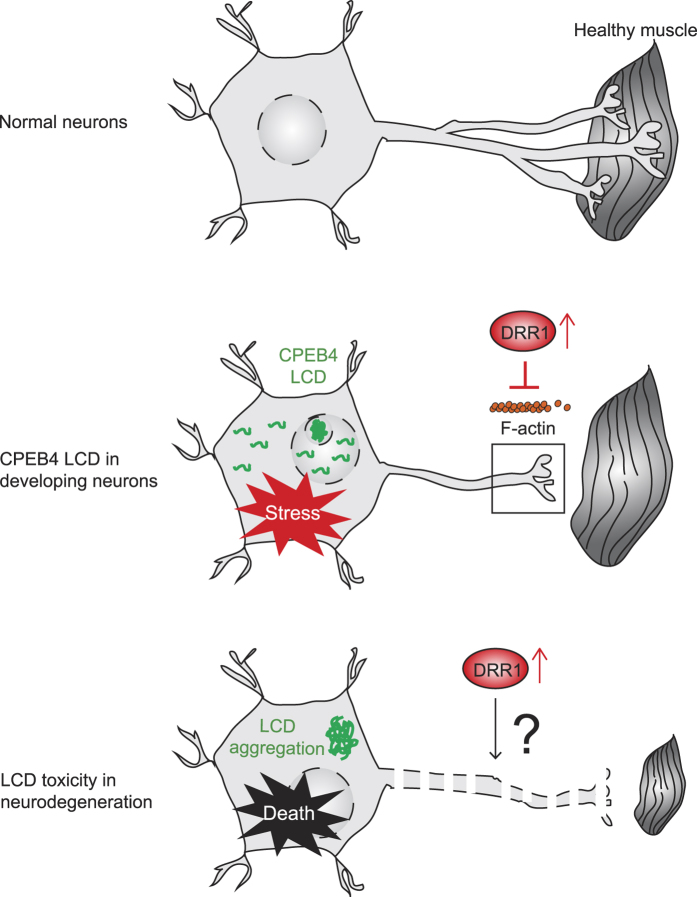
Proposed model for a convergent pathway of LCD-mediated toxicity. In normal neurons, neurites are well arborized and synapse to healthy muscles to form NMJ. In CPEB4 LCD (green) expressing developing neurons, CPEB4 LCD induces cellular stress as well as DRR1 (red circle) up-regulation, which inhibits F-actin (small orange circles) polymerization for neurite outgrowth in axons and growth cones (black rectangle). The consequences are defects in axon branching and NMJ formation (axon is not extended to muscles). In LCD-related neurodegeneration disorders such as ALS, LCD aggregates are generally localized in the cytoplasm (and in nucleoli in Kwon *et al*.^38^), which induces stress, DRR1 expression, and eventually neuronal cell death (indicated by fragmented axonal compartments and shrunken muscles).

DRR1 is an actin bundling protein^42^ detected in neuronal growth cones (Fig. 5C), which reinforces the notion that impedance of cytoskeleton dynamics could have dire consequences for various neuronal defects. These observations also support the notion that temporarily distinguishable groups of neurological disease may target a common pathological pathway. Therefore, we propose that CPEB4 LCD triggers a stress response that includes DRR1 upregulation along with other molecular changes, which subsequently contributes to impairment of neurite outgrowth and NMJ formation via disruption of actin organization (Fig. 6). The fact that elevated *Drr1* mRNA occurs in other neurological diseases suggests a potentially broad role of DRR1 and actin filament organization pathway in neuronal pathology for both development and degeneration.

## Discussion

We characterized a mouse model (*Cpeb4*^GT/GT^) that expresses truncated CPEB4 protein but no full-length protein. Because *Cpeb4* null mice (*Cpeb4*^ΔE2/ΔE2^) exhibit no overt phenotype^33^ (Fig. 2D), we speculate that key phenotypes of the *Cpeb4*^GT/GT^ animals presented in this study are exclusively due to expression of the LCD. The mobility deficits, impaired motor axon branching, abnormal NMJ formation, and altered molecular readouts of the *Cpeb4*^GT/GT^ embryonic and neonatal mice described in this study suggest a potential relationship between LC protein toxicity and neurodevelopmental disease. Accumulating evidence has clearly linked LCD-containing proteins to neurodegeneration through pathogenic mechanisms, in part, involving accelerated protein aggregation. This phenomenon is perhaps best exemplified by the polyglutamine oligomers seen in CAG repeat expansion diseases including HD, spinal bulbar muscular atrophy (SBMA), and spinocerebellar ataxia (SCA). The expanded polyglutamine peptides can assemble into large self-associated oligomers whose possible toxicity is correlated with disease. RAN translation products generated from microsatellite repeat expansions in several neurological diseases may be other illustrations of LCD toxicity. CAG repeats in SCA8, CTG repeats in myotonic dystrophy (DM) type 1, and CGG repeats in Fragile X tremor ataxia syndrome (FXTAS) all produce homopolymeric peptides that accumulate in human patient tissues^49^. ALS-causing GGGGCC repeats produce multiple poly-dipeptides, which produce toxic effects in cell cultures and animals^38^,^50^,^51^. In these and other cases, the precise cause of the toxicity remains elusive.

Interestingly, aberrant cleavage of protein fragments harboring LC region is often associated with several neurological diseases. For example, SCA3 is caused by abnormal expansion of the polyglutamine in the C-term region of the protein, and the cleavage of the C-term fragment by caspases has been suggested to be essential for pathology of the disease^52^. Similarly in HD, proteolytically cleaved Huntingtin fragments containing expanded polyglutamine repeats may be required to initiate toxicity related to disease neuropathology^53^. In addition, in the case of ALS, aberrant cleavage of TDP-43 C-term region containing disordered LCD contributes to enhance aggregation and cellular toxicity^54^,^55^. These and other observations suggest aberrant protein truncation and resulting expression of LCD peptides/proteins may be a general pathogenic mechanism for neurological disease.

The aberrant expression of CPEB4 LCD induces some forms of cellular stress, which is manifest by increased levels of a specific set of stress response genes and formation of nucleolar puncta and altered rRNA biogenesis, some of which are also seen in diseases such as SMA and ALS^45^,^48^,^51^,^56^. We have no evidence of neurodegeneration in fetal or adult *Cpeb4*^GT/GT^ mice (indeed, our data indicate lack of cell death), but we find that axon development in the embryo is impaired, which likely contributes to the high mortality rate in the few weeks after birth. Thus, we surmise that a recapitulation of CPEB4 LCD gain-of function toxicity in humans would be most likely to result in fetal or infant mortality.

One important finding of our study is that the actin-bundling protein DRR1 may be a target of neuron dysfunction. *Drr1* is one of a few mRNAs that is up-regulated in the spinal cords of *Cpeb4*^GT/GT^ mice and is likely to be at least partly responsible for impaired axogenesis (Fig. 5E–G). Originally found to be down-regulated in renal carcinoma, DRR1 is also thought to mediate malignant glioma invasion by reorganizing the actin cytoskeleton^57^,^58^. More importantly, DRR1 expression is specific to neuronal tissues (Supplemental Fig. S5A) and DRR1 is localized to growth cones of cultured hippocampal neurons (Fig. 5C), which are the mobile tips of developing neurites where the DRR1 protein co-localizes with actin filaments and promotes their stabilization^42^. When ectopically expressed, DRR1 partially abrogates actin-dependent neurite outgrowth, and viral-mediated ectopic expression in mouse brain CA3 region of the hippocampus alters cognitive performance as well as social interactions^42^,^44^. Additionally, *Drr1* is highly expressed in human outer radial glia, the neural stem cells of the neocortex, during cortical development, suggesting its importance in the coordination of neurodevelopment^59^. Taken together, these observations suggest an underappreciated role of DRR1 in neuron development, maintenance and maybe repair through regulating actin dynamics. One model, therefore, posits involvement of DRR1 up-regulation in LCD-mediated neurodegeneration as well as in impaired axon development (Fig. 6). While axons grow and synapse to healthy muscles in normal neurons, in case of *Cpeb4*^GT/GT^ mice, CPEB4 LCD protein induce cellular stress that increases DRR1 in axons and growth cones that impedes polymerization of F-actin, which inhibits neurite outgrowth and promotes axon thinning, defects in branching, and abnormal NMJ formation. CPEB4 LCD proteins localize not only diffusely to nucleoplasm and cytoplasm, but also to nucleolar puncta. The precise degree of pathogenic contribution from the nucleolar CPEB4 LCD aggregates is unclear even though perturbation of genes related to ribosome function (i.e., ribosomal protein encoding genes) was observed. In LCD-related neurodegeneration such as ALS, LCD aggregates are generally localized in cytoplasm and correlated with cellular stress (and eventual cell death) as well as increased DRR1. We propose that DRR1 may be an important contributing factor in degeneration, and thus could be a focal molecule for convergent pathways for various LCD-related neurological disorders.

In summary, we report that the CPEB4 LCD impairs neuron development that results in reduced motor axon branching and diminished NMJ formation in embryos and uncoordinated movement in neonatal mice. At least one important molecular lesion is the elevated expression of DRR1, whose actin bundling activity likely impedes neurite outgrowth. Heightened DRR1 levels appear to be a general feature of neuron pathology, and thus may be a common link among various neurological diseases. We propose molecular readouts of this mouse model may reveal important pathways that are widely involved in neurodevelopmental and neurodegenerative disease.

## Methods

### Mice

Animal protocols were approved for use by the University of Massachusetts Medical School Institutional Animal Care and Use Committee (IACUC). All experimental procedures were performed in accordance with the approved protocols. Heterozygous *Cpeb4* gene trap (*Cpeb4*^tm1a(EUCOMM)Wtsi^, EMMA ID:04002, *Cpeb4*^GT/+^) mice were obtained from European conditional mouse mutagenesis program. For motor neuron identification in spinal cord, *Cpeb4*^GT/+^ was bred to Hb9::GFP animals obtained from the Jackson Laboratory (B6.Cg-Tg(Hlxb9-GFP)1Tmj/J, stock number 005029). To generate *Cpeb4*^ΔE2/ΔE2^ null mice, *Cpeb4*^GT/+^ mice were bred to C57Bl6/J congenic ROSA26::FlpE knock-in mice and protamine Cre transgenic mice that were obtained from Dr. Gregory Pazour (University of Massachusetts Medical School).

### Genotyping

To obtain genomic DNA, tails snips were lysed in protease buffer (50 mM KCl, 10 mM Tris-HCl pH 8.3, 2.5 mM MgCl_2_, 0.45% NP-40, 0.45% Tween-20, 150 ng/μl proteinase K) at 60 °C for 45 minutes. PCR was performed with 35 cycles of 30″ at 95 °C, 30″ at 60 °C, and 30″ at 72 °C. GFP, FlpE, and Cre genotyping was done following the Jackson Lab protocol.

### Mouse behavior analysis

Body weight was measured with 2–3 animals at P0, 5–9 animals at P4, and 6–10 animals at P13. Gastric milk spots in p0 newborn *Cpeb4*^+/+^ (n = 13) or *Cpeb4*^GT/GT^ (n = 15) mice were observed mid-morning and categorized in three groups: absent, partially full, and full. For the nipple attachment test, the mother was anesthetized and laid with belly up and p0 *Cpeb4*^+/+^ (n = 9) or *Cpeb4*^GT/GT^ (n = 6) pups placed on the nipple. Each pup received help until the moment it was successfully attached, and time for attachment was recorded. 300 seconds was the maximum duration of the test.

### RNA extraction and RT-PCR

Trizol-extracted total RNA was used for reverse transcription (1–5 ug of RNA) with Super Script III (Invitrogen). PCR with cDNA was performed routinely using 2x GoTaq (Promega).

### Whole mount staining

For X-gal staining, whole embryos or dissected tissues were fixed in 0.2% glutaraldehyde, 2% formalin, 5 mM EGTA, and 2 mM MgCl_2_ in 0.1 M phosphate buffer and stained with 1 mg/ml x-gal, 5 mM potassium ferricyanide, and 5 mM potassium ferrocyanide. After washing with PBS, they were photographed and stored in 80% ethanol at 4 °C. *Cpeb4*^GT/+^ tissues were always stained together with *Cpeb4*^+/+^ littermates to serve as controls. Whole-mount NF staining for mouse diaphragm was performed as described^60^ using 2H3 antibody (Developmental studies hybridoma bank), and right ventral region of the diaphragm was photographed. The diaphragm was also co-stained with Alexa Fluor 594 conjugated α-bungarotoxin (Life science) to visualize NMJs. Axon tract tracing was performed using ImageJ plugins, MosaicJ and NeuronJ.

### Immunostaining for spinal motor neurons

Freshly harvested mouse spinals cords were fixed with 4% paraformaldehyde overnight, immersed in 30% sucrose/PBS, and embedded with OCT. 20 μm thick serial sections were prepared for every 200 μm. Cryosections were blocked with blocking solution (3% BSA, 5% goat serum in PBS with 0.1% Triton X-100). Sections were then incubated with anti-GFP antibody (Abcam) followed by FITC-conjugated secondary antibody. Binary images of GFP positive area were made to calculate area for motor neurons in each section.

### Microarray gene expression analysis

Spinal cords from several p0 *Cpeb4*^+/+^ or *Cpeb4*^GT/GT^ were pooled to reduce individual variability and subjected to Illumina MouseRef-8 BeadChip microarray assays containing approximately 18,000 RefSeq transcripts. Further analysis on duplicates of samples was performed by using Agilent GeneSpring GX software. 8122 RefSeq genes were detected above threshold (expression level > 100), 1022 (12.58%) of which were differentially expressed in *Cpeb4*^GT/GT^ tissue (paired t-test, p < 0.1). For gene ontology analysis, DAVID functional annotation tool (http://david.abcc.ncifcrf.gov/) was used. A complete list of differentially expressed genes is in Supplemental Table 1.

### Quantitative real-time PCR

Real time PCR was performed with an ABI 7300 instrument with Maxima SYBR green with ROX (Fermentas). For quantification of gene expression, the ΔΔCt method was used normalizing to *Ppia*.

### Recombinant protein and antibody purification

A CPEB4 N-term 188 residue fragment was cloned into pET28a to generate His-tagged recombinant protein, which was purified on Ni-NTA resin (Thermo Scientifics) and used for CPEB4 antibody affinity purification.

### Lentiviral vector cloning

FLAG tagged *Drr1* was amplified and ligated into pFUGW lentiviral expression vector using BamHI and EcoRI sites. For knockdown of *Drr1*, gene specific oligonucleotides were annealed and inserted into the pll3.7-Syn lentiviral shRNA vector.

### Cell culture, transfection, and lentivirus transduction

The culture and maintenance of primary spinal cord^61^ and hippocampal neurons were performed as described^62^ in neurobasal medium containing 2% B27 and 1% Glutamax. HEK293T and N2a cells were routinely cultured using 10% FBS and 1% antibiotic, antimycotic in DMEM (Gibco). Triple transfection for virus production was performed as follows: for 80% confluent HEK293T cells in 10 cm dish, 5 μg lentiviral vector, 2.5 μg pMD2.G, and 4 μg psPAX2 was used with 20 ul Lipofectamine 2000. After 72 hours, the supernatant was collected, filtered and used for transduction.

### Subcellular fractionation and localization

Subcellular fractionation was done as previously described^63^. Fractionated proteins were subjected to western blot analysis using anti-lamin A/C (Cell Signaling), anti-H3 (Abcam), and anti-alpha tubulin (Sigma).

### Immunocytochemistry

Cells were grown on coverslips, fixed with 4% paraformaldehyde (PFA), blocked with blocking solution, immunostained with GFP (Abcam) or FUS (Bethyl lab) antibody. The Z stack maximum projection images were obtained using ImageJ software. For detection of nuclear puncta, all stacks were individually examined. All microscope settings were kept constant for all images taken in one experiment.

### Sholl analysis

Hippocampal neurons were grown on poly-L-lysine (Sigma) coated coverslips for 4–7 days, fixed in 4% PFA, and stained with anti-alpha tubulin (Sigma) to visualize neurites. Sholl analysis was performed as described^64^.

### Statistics

In all panels, one asterisk indicates p < 0.05, two asterisks indicate p < 0.01, and three asterisks indicate p < 0.001 (Student’s t test).

### Analysis of NCBI Gene Expression Omnibus (GEO) data sets

The concept of using publically available gene expression microarray studies for neurodegenerative diseases and the method for data collection was adapted from a published study^45^. The processed gene expression data sets for seven different gene expression microarrays were downloaded and analyzed using GEO2R available at http://www.ncbi.nlm.nih.gov/geo/geo2r. The log_2_ fold change values for *Drr1*, *Nfkbia*, and *Edn1* were obtained to generate a heat map for visualization of the expression level changes in the neurodegenerative diseases. Data sets for *Smn*-/-;SMN2 mouse (fold change >1.5 for p5 spinal cord) and schizophrenia/bipolar patients were obtained from the literature^47^,^48^.

## Additional Information

**How to cite this article**: Shin, J. *et al*. Impaired neurodevelopment by the low complexity domain of CPEB4 reveals a convergent pathway with neurodegeneration. *Sci. Rep.* **6**, 29395; doi: 10.1038/srep29395 (2016).

## Supplementary Material

Supplementary Information

Supplementary Table S1

Supplemental Movie S1

Supplemental Movie S2
