# Supplementary material for: Impaired neurodevelopment by the low complexity domain of CPEB4 reveals a convergent pathway with neurodegeneration
Source: Sci Rep. 2016 Jul 6;6:29395. doi: 10.1038/srep29395 (PMC4933966; doi:10.1038/srep29395)
Supplement: Supplementary Information [file srep29395-s1.pdf]

# Impaired neurodevelopment by the low complexity domain of CPEB4 reveals a convergent pathway with neurodegeneration

Jihae Shin[1], Johnny S. Salameh[2], and Joel D. Richter[1*]

[1]Program in Molecular Medicine

[2]Department of Neurology

University of Massachusetts Medical School

# Figure S1, related to Figure 2

A

1 MGDYGFGVLVQSNTGNKSAFPVRFHPHLQPPHHHQNATPSPAAFINNNTAANGSSAGSAW
61 LFPAPATHNIQDEILGSEKAKSQQQEQQDPLEKQQLSPSPGQEAGILPETEKAKSEENQG
121 DNSSENGNGKEKIRIESPVLTGFDYQEATGLGTSTQPLTSSASSLTGFSNWSAAIAPSSS
181 TIINEDASFFHQGGVPAASANNGALLFQNFPHHVSPGFGGSFSPQIGPLSQHHPHHPHFQ
241 HHHSQHQQQRRSPASPHPPPFTHRNAAFNQLPHLANNLNKPPSPWSSYQSPSPTPSSSWS
301 PGGGGYGGWGGSQGRDHRRGLNGGITPLNSISPLKKNFASNHIQLQKYARPSSAFAPKSW
361 MEDSLNRADNIFPFP

B

| AA | # | % |
|---|---|---|
| S | 50 | 13.3 |
| P | 41 | 10.9 |
| G | 35 | 9.3 |
| A | 32 | 8.5 |
| Q | 29 | 7.7 |
| N | 28 | 7.5 |
| SUM | 215 | 57.3 |

C

| | This study | Hu et al. (2014) | Tsai et al. (2013) |
|---|---|---|---|
| Cpeb4 GT/GT | C57bl/6NTac X C57bl/6 | C57bl/6NTac X C57bl/6 | N/A |
| Cpeb4 ΔE2/ΔE2 | C57bl/6NTac X C57bl/6 | N/A | C57bl/6 |
| FlpE | C57bl/6 | N/A | N/A |
| Protamine Cre | C57bl/6 | N/A | C57bl/6 |

# Figure S2, related to Figure 3

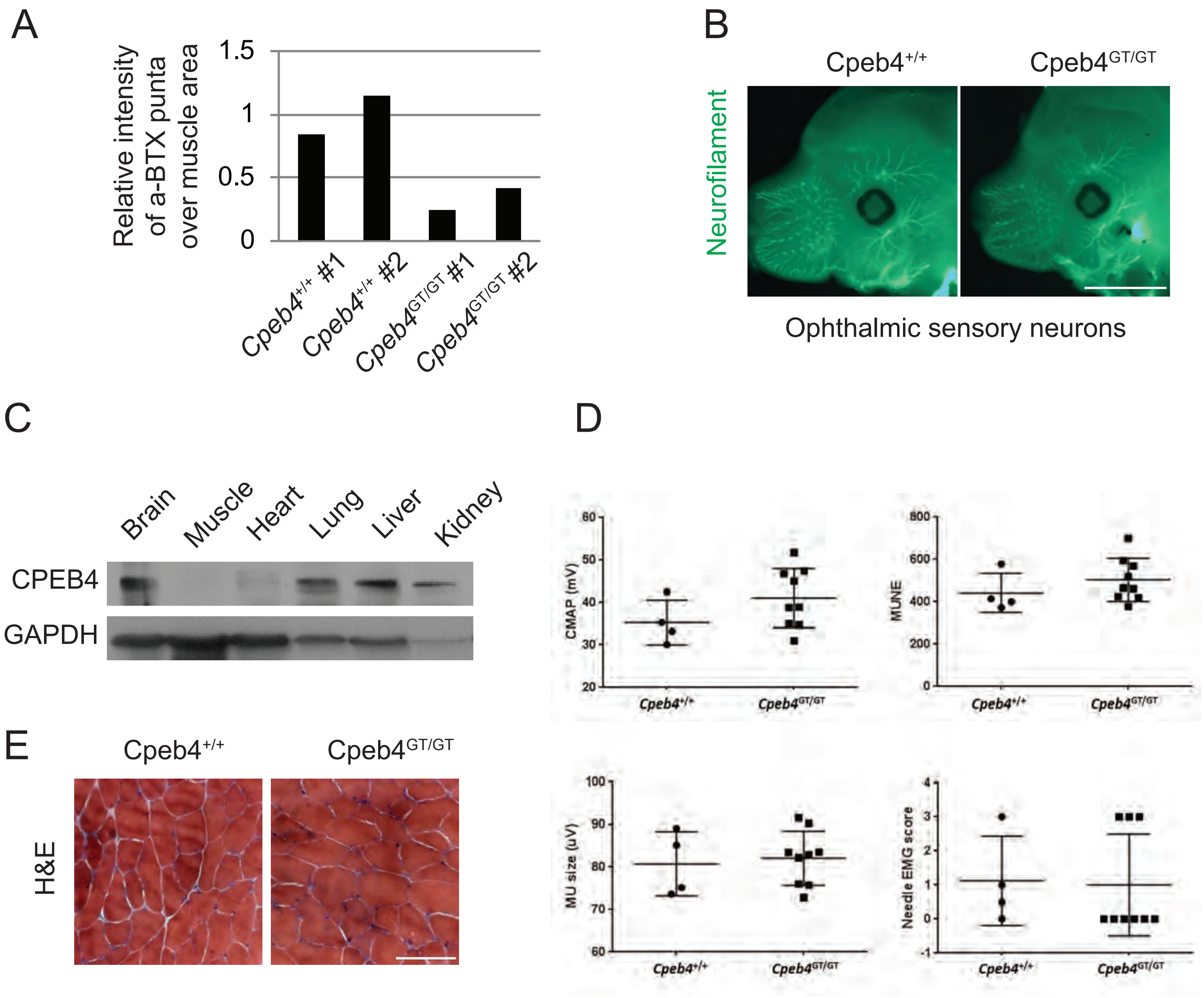

# Figure S3, related to Figure 4

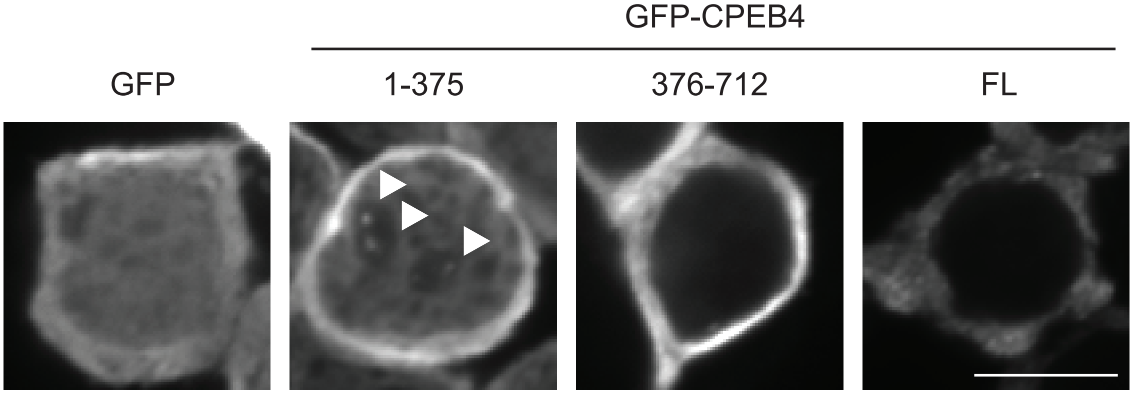

# Figure S4, related to Figure 5

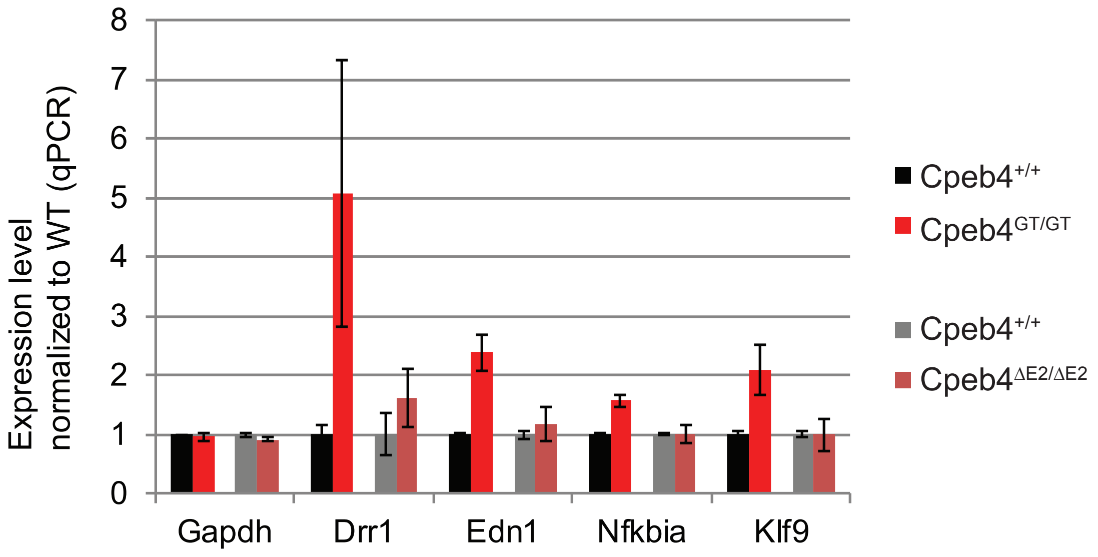

# Figure S5, related to Figure 5

A

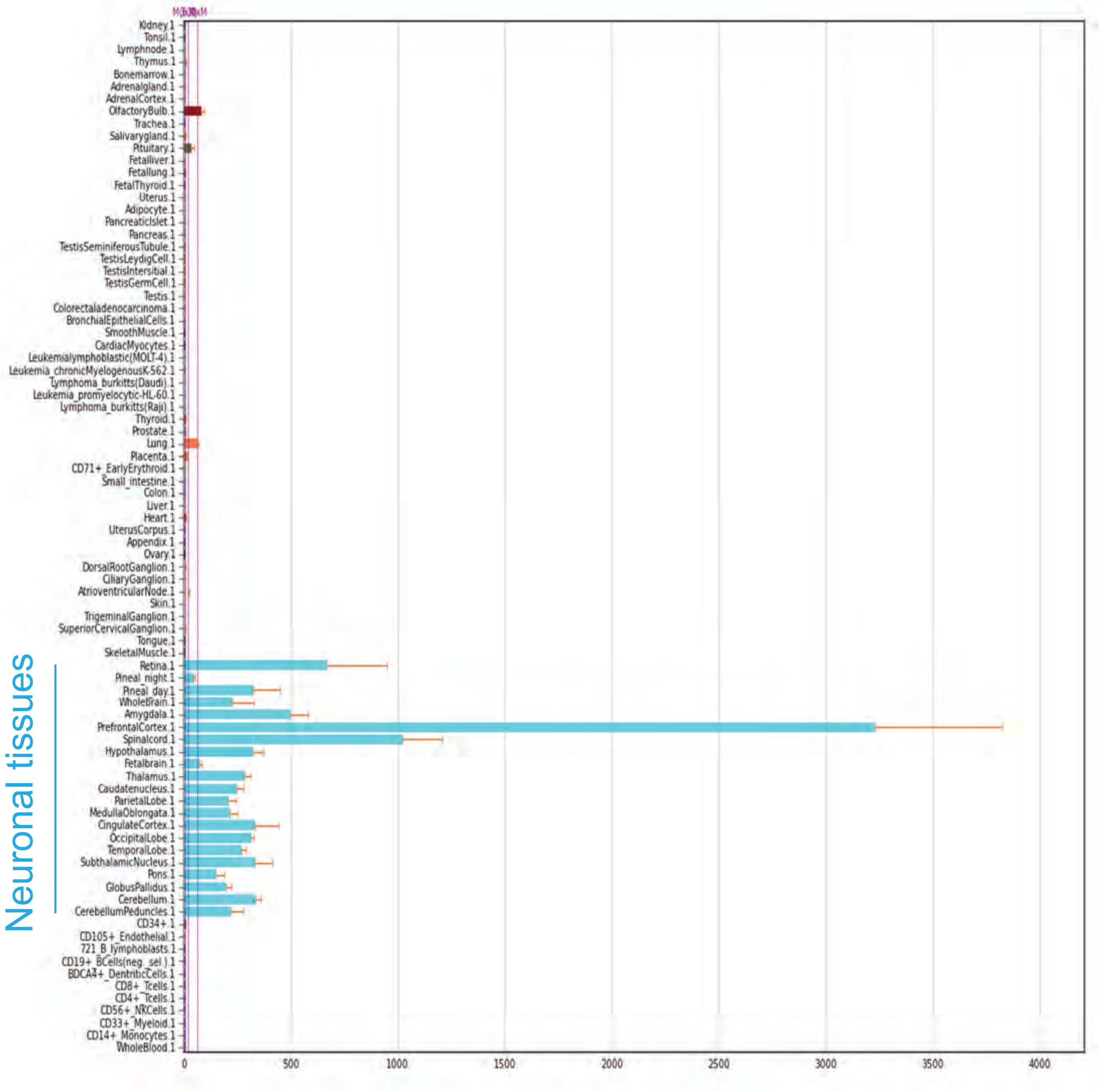


B

| Reference | Disease | Accession # | Original study first author | Year | Species | Tissue Source |
|---|---|---|---|---|---|---|
| Li (2014) | ALS | GSE26927 | Durrenberger | 2012 | Human | Cervical spinal cord |
| | ALS | GSE4595 | Lederer | 2007 | Human | Motor cortex |
| | HD | GSE26927 | Durrenberger | 2012 | Human | Ventral head of caudate nucleus |
| | PD | GSE26927 | Durrenberger | 2012 | Human | Substantia nigra |
| | PD | GSE7621 | Lesnick | 2007 | Human | Substantia nigra |
| | PD | GSE20333 | Grunblatt | 2004 | Human | Substantia nigra |
| | PD | GSE20164 | Hauser | 2005 | Human | Substantia nigra |
| Shao (2008) | SZ | N/A | Shao | 2008 | Human | Dorsolateral prefrontal cortex |
| | BPD | N/A | Shao | 2008 | Human | Dorsolateral prefrontal cortex |
| Murray (2010) | SMA | N/A | Murray | 2010 | Mouse | Spinal cord |

Supplemental legends

Movie S1. Suckling behavior and mobility during nipple attachment test in *Cpeb4*$^{+/+}$ and *Cpeb4*$^{GT/GT}$ mouse pups at p0.

Movie S2. Gasping of *Cpeb4*$^{GT/GT}$ pup at p0 (right).

Table S1. Differentially regulated genes in p0 *Cpeb4*$^{GT/GT}$ mouse spinal cord.

Figure S1, related to figure 2. (A) Protein sequence of CPEB4 exon 1 and the amino acid composition. (B) The number and percentage of amino acid usage is summarized. (C) Genetic background of the mice used in this study, Hu et al. (2014) and Tsai et al. (2013).

Figure S2, related to figure 3. (A) Quantification of a-BTX puncta area over the muscle area of *Cpeb4*$^{+/+}$ and *Cpeb4*$^{GT/GT}$ diaphragms (scale relative to mean of WT). (B) Whole mount anti-neurofilament staining of e12.5 mouse face for ophthalmic sensory axon branching. (C) Western blot analysis of various mouse tissues probed with CPEB4 antibody. GAPDH is a loading control. (D) Electromyography (EMG) for sciatic nerve of 2 year old *Cpeb4*$^{+/+}$ and *Cpeb4*$^{GT/GT}$ mice to measure motor neuron degeneration. CMAP, measurement of compound muscle action potential. MUNE, motor unit number estimation. MU, motor unit. (E) H&E histology of tibialis anterior muscles in *Cpeb4*$^{+/+}$ and *Cpeb4*$^{GT/GT}$ mice.

Figure S3, related to figure 4. N2a cells transfected with GFP, GFP-CPEB4 residue 1-375, GFP-CPEB4 376-712, or GFP-CPEB4 full length (FL) were stained with GFP and FUS (nuclear protein) antibodies. These are magnified images of Fig. 4D.

Figure S4, related to figure 5. Quantitative RT-PCR of *Drr1*, Edn1, *Klf9,* and *Nfkbia* RNAs normalized to *Ppia* mRNA from p0 *Cpeb4*$^{+/+}$ (gray) and *Cpeb4*$^{\Delta E2/\Delta E2}$ (grayish red) spinal cord (mean ± SEM). Histograms for *Cpeb4*$^{+/+}$ (black) and *Cpeb4*$^{GT/GT}$ (red) were duplicated from Fig. 5B to show the relative expression levels.

Figure S5, related to figure 5. (A) Expression level of *Drr1* in various human tissues from BioGPS (http://biogps.org); GeneAtlas U133A, gcrma; probeset is 207547_s_at. (B) Summary of public gene expression data sets used in Fig. 5H.

Supplemental Experimental Procedures

Oligonucleotides sequences

| | | |
|---|---|---|
| $Cpeb4^{GT/+}$ | fwd | 5’-AGGACGTTTGACATGCACTCACTGGAGAGCTCACT-3’ |
| $Cpeb4^{GT/+}$ | rev | 5’-CCTGCTTATATTCTGGCCTCAAGTTTGTTAGTGACTATGTT-3’ |
| $Cpeb4^{\Delta E2/\Delta E2}$ | fwd | 5’-CATAATTCATAGAGAGTGGAGGTCCTATCAAAATCAC-3’ |
| Cpeb4 exon 1 | rev | 5’-CATGTCAGCCCTGGCTTTGGT-3’ |
| Cpeb4 exon 6 | rev | 5’-TGGTTTATCCTTGATGGTTGGACTTG-3’ |
| Cpeb4 exon 10 | rev | 5’-CATTCTTCAGTTCCAGCGGAATG-3’ |
| Drr1 | fwd | 5’-GTAGGACCACCCAAGGGACTTAGC-3’ |
| Drr1 | rev | 5’-GGGACTAACAGAGTCACAGAGTATCAAGGT-3’ |
| Gapdh | fwd | 5’-AACGACCCCTTCATTGACCT-3’ |
| Gapdh | rev | 5’-TGGAAGATGGTGATGGGCTT-3’ |
| FLAG tagged Drr1 | fwd | 5’- GCCTCCGGATCCAGCCTCCATGTACTCAGAGA-3’ |
| FLAG tagged Drr1 | rev | 5’-CGGTCCGGAATTCCTTGCTCACTTGTCATCGTCG TCCTTGTAGTCCAGTGCTCTTTCCTCGCTGGT-3’ |
| shRNA Drr1 | fwd | 5’-TGGCTGACATCGAGGGACTTTCAAGAGA-3’ |
| shRNA Drr1 | rev | 5’-TCGAGAAAAAAGGGCTGACATCGAGGGA-3’ |
| Ppia | fwd | 5’-CTCCTCGAGCTGTTTGCAGACAAAG-3’ |
| Ppia | rev | 5’-ACCCTGGCACATGAATCCTGGAA-3’ |
| Edn1 | fwd | 5’-AGGCCATCAGCAATAGCATC-3’ |
| Edn1 | rev | 5’-TTGTGCGTCAACTTCTGGTC-3’ |
| Klf9 | fwd | 5’-CACATGGAAATCTGCCACAG-3’ |
| Klf9 | rev | 5’-CAAGGGGACCAAATGTTGAC-3’ |
| Nfkbia | fwd | 5’-ACCTGGTTTCGCTCTTGTTG-3’ |
| Nfkbia | rev | 5’-TGGAGATTTTCCAGGGTCAG-3’ |
| 45S | fwd | 5'-GAGCTGGTGGTGGCGCTCC-3' |
| 45S | rev | 5'-CTTGCCCCTCCTTCTCT-3' |
| 18S 5junction | fwd | 5'-GACGCTCCGCTCGCGCTTCCTTACCT-3' |
| 18S 5 junction | rev | 5'-TAGACATGCATGGCTTAATCTTTG-3' |
| 18S 3 junction | fwd | 5'-AGTCGTAACAAGGTTTCCGTAGGT-3' |
| 18S 3 junction | rev | 5'-CCACAGTCTCCCGTTTAAT-3' |
| 5.8S 5junction | fwd | 5'-TACGACTCTTAGCGGTGGATCA-3' |
| 5.8S 5junction | rev | 5'-TCACATTAATTCTCGCAGCTAGCT-3' |
| 5.8S 3junction | fwd | 5'-GAATTGCAGGACACATTGATCATC-3' |
| 5.8S 3junction | rev | 5'-GTCAACCGACGCTCAGA-3' |
| 28S 5junction | fwd | 5'-CTGACCGCGACCTCAGAT-3' |
| 28S 5junction | rev | 5'-TCCGCTGACTAATATGCTTAAATTCA-3' |
| Mature 18S | fwd | 5'-GATGGTAGTCGCCGTGCC-3' |
| Mature 18S | rev | 5'-GCCTGCTGCCTTCCTTGG-3' |
| Mature 5.8 | fwd | 5'-ACTCGGCTCGTGCGTC-3' |
| Mature 5.8 | rev | 5'-CCGACGCTCAGACAGG-3' |
| Mature 28S | fwd | 5'-GACGCGCATGAATGGA-3' |
| Mature 28S | rev | 5'-TGTGGTTTCGCTGGATAGTAGGT-3' |

## Electrophysiological recordings

Electrophysiological recordings were done as previously described (Xia et al., 2010) on 2 year old mice. Mice were deeply anesthetized with isoflurane. Motor conduction studies and motor unit number estimate (MUNE) were performed using a portable electrodiagnostic system (Cardinal Synergy). For the MUNE recordings, the incremental technique was used (Shefner et al., 2006). After recording from each side, the animals were euthanized.

## Quantification of a-BTX puncta

Images for whole mount diaphragm were stacked for maximum intensity and binary images of a-BTX positive area were analyzed for particle area (ImageJ).

Supplemental References

Shefner, J.M., Cudkowicz, M., and Brown, R.H., Jr. (2006). Motor unit number estimation predicts disease onset and survival in a transgenic mouse model of amyotrophic lateral sclerosis. Muscle & nerve *34*, 603-607.

Xia, R.H., Yosef, N., and Ubogu, E.E. (2010). Dorsal caudal tail and sciatic motor nerve conduction studies in adult mice: technical aspects and normative data. Muscle & nerve *41*, 850-856.
